# The preliminary molecular study of four skink species in Rajaji Tiger Reserve (RTR), Uttarakhand, using 12S rRNA mitochondrial locus

**DOI:** 10.1080/23802359.2017.1357435

**Published:** 2017-08-02

**Authors:** Ankita Rajpoot, Archana Bahuguna, Ved Prakash Kumar, Dhyanendra Kumar

**Affiliations:** a Zoological Survey of India, NRC, Dehradun, India;; b Wildlife Forensic and Conservation Genetics Cell, Wildlife Institute of India, Dehradun, India;; c Veer Kunwar Singh University, Arrah, India

**Keywords:** *Eutropis macularia*, *Eutropis carinata*, *Asymblepharus himalayanus*, *Lygosoma punctata*, mtDNA, 12S rRNA, conservation

## Abstract

Skinks are present under the Scincidae family, widely distributed species in Indian subcontinent. Uttarakhand is one of the hotspot where number of identified and unidentified skink species reported. Herein, we first time provided the 12S rRNA genetic reference database of four skink species, i.e. *Eutropis macularia*, *Eutropis carinata*, *Asymblepharus himalayanus* and *Lygosoma punctata*, in Rajaji Tiger Reserve (RTR), Uttarakhand (India). The identified four species belong to three different genera, where *Eutropis carinata* and *Asymblepharus himalayanus* listed Least Concern and Vulnerable in IUCN, respectively. Here, we collected tissue samples of four different skink species from Rajaji Tiger Reserve during field survey. After successful laboratory procedure, we compared obtained sequences with publically available genetic database and we observed four sequences matched with respective species. Furthermore, the evolutionary sequence divergence result revealed that the *Eutropis carinata* and *Eutropis macularia* are close to each other with 0.11 genetic distance. The present study indicates that the exact number and population distribution of skink species are unidentified; therefore, herein we suggest the proper screening of Uttarakhand population around should be investigated, further genetic study in combination with a good sampling strategy to investigate species biology and status for conservation program.

## Introduction

The family Scincidae (skinks) is the largest from the sixteen families of lizards with more than 1500 reported species (Mecke et al. 2013; Uetz et al. 2013). Scincidae (skinks) belong to the infraorder Scincomorpha, a monophyletic group of families whose members tend to be elongate and have relatively long-snouted and somewhat flattened skulls, in which the upper temporal opening is usually reduced or lost. One of every four species of lizard is a skink, making them a significant component of reptile diversity. Skinks are most diverse group of lizards that contains terrestrial species (active on the surface of the ground or low perches) a significant number are secretive to fossorial (carrying out most activities within leaf litter or underground) and arboreal species (climbers, living in trees or on rocks). Skinks are typically diurnal but some species are nocturnal. Furthermore, it found in all types of environments, from tropical forests to desert, savannas, lowland rainforests, temperate forests and cool mountain habitats (Daniel 2002). Recently, a seven-family taxonomic scheme proposed to replace formal and informal group names for skinks that have become widely used over the years (Hedges and Conn 2012). However, skinks are usually placed in a single family, Scincidae (Oppel 1811).

In India, there are several molecular studies that have been done on different groups of Scincidae family, such as genus Lygosoma and Eutropis (Venugopal 2010; Dutta-Roy 2012, 2014). According to recent studies, India harbours diverse faunas of reptiles nearly 518 species which includes 3 species of crocodiles, 34 species of turtles and tortoises, 202 species of lizards and 279 species of snakes belonging to 28 families recorded (Murthy 2010; Aengals et al. 2011). Approximately 58 species of skinks (family Scincidae) are widely distributed in Indian subcontinent (Venugopal 2010; Aengals et al. 2011; Bahuguna 2010).

Currently, the knowledge of distribution and status is unknown due to lack of research study. Skinks are excellent dispersers, many having rafted across oceans to colonize other continents and even remote islands in the Pacific. The head is usually covered with enlarged plates, termed head shields, and osteoderms are frequently present in some or all scales. Typically, skinks are slightly to markedly elongate lizards with moderate to short limbs and glossy cycloid scales, reinforced by characteristic compound osteoderms. In Uttarakhand, nine species belonging to five families of lizards have been reported by Chopra (1995) from Rajaji Tiger Reserve (RTR) and also reported by many workers from conservation areas of the state as well as from various districts of Uttarakhand.

The present study is a small part of our research conducted in our field area RTR, Uttarakhand (India), in 2017. The RTR is an Indian reserve that encompasses the Shivaliks, near the foothills of the Himalayas and the Indo-Gangetic plains recently declared as Tiger Reserve and its boundary extend to 1075 km^2^ from 820 km^2^. The RTR is spread over 1075 km^2^ and three districts of Uttarakhand: Haridwar, Dehradun and Pauri Garhwal. The vegetation of RTR is broadleaved deciduous forests, riverine vegetation, and scrubland, grasslands and pine forests. This type of habitat is favourable for mammals, reptiles, insects and plant species. Six skink species were sited during field survey, i.e. *Eutropis macularia* or little skink (Blyth 1854), *Eutropis carinata* or bronze grass skink (Schneider 1801*)*, *Eutropis dissimilis* or striped grass skink (Hallowell 1857*), Asymblepharus himalayanus* or Himalayan ground skink *(*Günther 1864), *Lygosoma punctata* or spotted supple skink (Gmelin 1799), and *Eurylepis taeniolata* or yellow belled Skink (Checklist India Biodiversity Portal 2015). Although we collected four species out of six remaining two *(Eutropis dissimilis* and *Eurylepis taeniolata*), sample was not possible due to low abundant. The single species such as *Eutropis carinata* and *Asymblepharus himalayanus* has been listed as Least Concern and Vulnerable in IUCN (De Silva and Vyas 2010) and remaining four not evaluated due to lack of population status and distribution.

Although until there are no any molecular studies conducted on RTR skinks population, therefore here we did preliminary molecular study by using partial fragment of 12S ribosomal RNA (350 bp) (Kocher et al. 1989) mitochondrial locus. Mitochondrial 12S rRNA mostly used for the species identification and deep phylogeny due to highly conserved characteristic and shows more difference between the interspecies (Kocher et al. 1989; Kumar et al. 2014; Rajpoot et al. 2016).

## Materials and methods

### Field survey and sample collection

We collected four different skink species from three different regions of RTR, during field survey conducted in 20–30 January 2017. During field survey, we sited number of individual but only collected the single individual per skink species. The flora and fauna of Tiger Reserve protected under the Indian Wildlife (Protection) Act, 1972; therefore, only collected tail part and species again release in field. To cross-verify our data, we also blast the quarry sequences by using the BLAST search tool implemented by National Centre for Biotechnology Information (NCBI, USAI) (http://blast.ncbi.nlm.nih.gov/) and also downloaded the same species reference sequences from the GenBank. Details of samples’ location and species given in are given in Table 1.

**Table 1.** Details of used skink species from present study and other sequences retrieved from GenBank for comparison.

Species	Genus	Origin	Accession no.	Conservation status
*Eutropis carinata*	*Eutropis*	RTR	MF370879	Least Concern version 3.1
*Eutropis macularia*	*Eutrois*	RTR M	MF370880	Not Evaluated
*Lygosoma punctata*	*Lygosoma*	RTR	MF370881	Not Evaluated
*Asymblepharus himalayanus*	*Asymblepharus*	RTR	MF370882	Vulnerable
Sequences retrieved from GenBank				
*Eutropis carinata*	*Eutropis*	GenBank	JQ767968.1	Least Concern version 3.1
*Eutropis macularia*	*Eutropis*	GenBank	AY070335.1	Not Evaluated
*Lygosoma punctata*	*Lygosoma*	GenBank	KF577788.1	Not Evaluated
*Asymblepharus himalayanus*	*Asymblepharus*	GenBank	KF514643.1	Vulnerable

### Laboratory procedure

The four tissue samples (tail) undertaken for the study were subjected before DNA extraction, rinsed these samples two times in 10 × phosphate-buffered saline (PBS) (kept for at least 20 min. on a shaking platform during each wash) and extracted genomic DNA using DNeasy Blood and Tissue Kit (Qiagen, Hilden, Germany) following the manufacturer’s protocol. The DNA concentration was quantified by absorbance measurement at 260 nm. Absorbance measurements at 260 nm were done using NanoDrop ND-1000 (Thermo Fisher Scientific Inc., Waltham, MA) with 1 μl of samples.

We successfully amplified partial fragments of the 12S rRNA locus (Kocher et al. 1989) in four samples. All PCRs were carried out on a same condition on Thermal Cycler 3700 (Quanta Biotech, Beverly, MA). Each reaction of 10 μl reaction contained 1 × PCR buffer (50 mM KCl, 10 mM Tris–HCl and 2.5 Mm MgCl_2_), 200 μM of each dNTP, 1.25 μg of BSA, 4 pM of each primer (forward and reverse) and 0.5U of Taq DNA polymerase (GeNei, Bangalore, India) and approximately 35–45 ng of genomic DNA. We set up cycling condition according to previously published condition (Rajpoot et al. 2016). On completion of PCRs, we electrophoresed PCR product on 1.5% agarose gel and visualized over transilluminator to detect the amplification. Positive and negative controls performed throughout all DNA extraction and PCR amplifications for monitoring any contamination. The sequencing of amplified product did thorough commercially available DNA sequencing facility (Merck, Bengaluru, India).

### Sequences cleaning and analysis

Quality of sequences was determined using Sequence Analysis v5.2 software (Applied Biosystems, Foster City, CA). Apart from that CLUSTALW algorithm implemented in BioEdit version 7.0.5.3 (Hall 1999) was used in the multiple sequence alignments (MSA). The sequences obtained from the four different species were compared with sequences available on NCBI through a BLAST search (http://blast.ncbi.nlm.nih.gov/) (Table 1). For the species diagnosis, we considered the percentage similarity between query and reference sequence pairs. To confirm MSA result, we compare our data with phylogenetic analysis conducted using MEGA version 7.0 (Kumar et al. 2016) for 12S rRNA using the neighbor-joining (NJ) method (Saitou and Nei 1987). Estimates of evolutionary divergence over sequence pairs between groups determined by using the Kimura 2-parameter distance (Kimura 1980) as implemented in MEGA version 7.0 (Kumar et al. 2016).

## Results and discussion

The obtained genomic DNA concentration in all samples was good and ranged from 10.90 ng/uL to 12.84 ng/uL. In PCR, a used mitochondrial locus was successfully amplified in all four skink species. The sample yielded readable sequences of length *ca* 330 bp. The CLUSTALW analysis with our sequences from present study and four other sequences retrieved from GenBank revealed that average nucleotide frequencies were 21.7 [T], 25.2 [C], 32.3 [A] and 20.9 [G]. The nucleotide ‘A’ showed the more number of nucleotide frequencies within four skink species (Table 2). Furthermore, the *ca* 330 bp 12S rRNA locus sequences consisted with 224 conserved regions (C), 107 variable sites (V), 95 parsimony informative sites (Pi) and 12 singleton sites (S) (Table 3). Furthermore, in four species n-81, fixed species-specific single nucleotide polymorphisms (SNPs) observed and out of these (n-81) SNPs, 15, 4, 29 and 33 were present in *Eutropis carinata, Eutropis macularia, Lygosoma punctata* and *Asymblepharus himalayanus*, respectively.

**Table 2.** Nucleotide frequencies in 12S rRNA sequences among four skink species. All frequencies are given in percent.

Species	T (U)	C	A	G	T
*Eutropis carinata* (RTR)	21.9	25.2	32.5	20.4	329
*Eutropis carinata*( JQ767968.1)	21.0	25.8	32.8	20.4	329
*Eutropis macularia* (RTR)	24.3	22.5	33.4	19.8	329
*Eutropis macularia* (AF153557.1)	23.9	23.9	32.7	19.4	330
*Lygosoma punctata* (RTR)	23.2	23.5	30.6	22.6	310
*Lygosoma punctata* (KF577788.1)	22.9	24.2	31.0	21.9	310
*Asymblepharus himalayanus* (RTR)	17.9	28.0	32.5	21.6	329
*Asymblepharus himalayanus* (KF514643.1)	18.2	28.3	32.2	21.3	329
Avg.	21.7	25.2	32.3	20.9	324.4

**Table 3.** Most similarities in the 12S rRNA based on NCBI GenBank BLAST search.

Species	Species with the highest similarity	Query coverage (%)	Similarity (%)	Overall			
*Eutropis carinata*	*Eutropis carinata*	100	99	C	V	Pi	S
*Eutropis macularia*	*Eutropis macularia*	100	95	224	107	95	12
*Lygosoma punctata*	*Lygosoma punctata*	100	99				
*Asymblepharus himalayanus*	*Asymblepharus himalayanus*	100	99				

**Figure 1.**
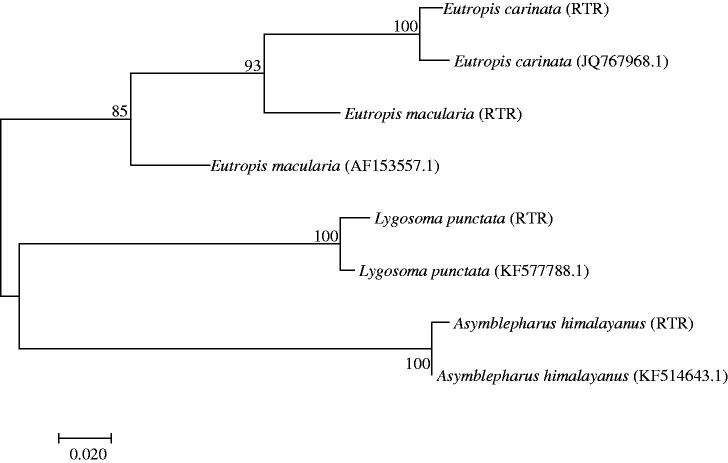
12S rRNA sequence based on neighbor joining relationship among four skink species. The optimal tree with the sum of branch length = 0.51519657 is shown. The percentage of replicate trees in which the associated taxa clustered together in the bootstrap test (1000 replicates) is shown next to the branches. The evolutionary distances were computed using the Kimura 2-parameter method and are in the units of the number of base substitutions per site. All positions containing gaps and missing data were eliminated. Evolutionary analyses were conducted in MEGA version 7.0.

To the species confirmation, sequences obtained were submitted as individually entries in BLAST analysis, 12S rRNA mtDNA locus indicated that the all samples were 95–99% similarity with respective skink species. Further, to cross-verify our result, analyzed obtained sequences with neighbor-joining topology, the neighbor-joining phylogenetic tree showed similar result (Figure 1). These four skink species belong to three different genera and tree topology clearly resolves the tree according to genus characteristic. The *Eutropis carinata* and *Eutropis macularia* present in same clade with 93% bootstrap value, while *Lygosoma punctata* and *Asymblepharus himalayanus* present with 85% value. All generated sequences submitted in GenBank and assigned accession numbers are given in Table 1 for future use.

The average genetic difference among four species was 0.020 and observed evolutionary divergence over sequence pairs between four species ranged from 0.011 to 0.30. The minimum sequence divergences (0.11) observed between *Eutropis carinata* and *Eutropis macularia,* while the maximum sequence (0.30) divergences observed between *Lygosoma punctata* and *Asymblepharus himalayanus* (Table 4).

**Table 4.** Estimates of evolutionary divergence over sequence pairs between groups.

Species	*Eutropis carinata*	*Eutropis macularia*	*Eutropis macularia*	*Asymblepharus himalayanus*
*Eutropis carinata*		0.02	0.03	0.03
*Eutropis macularia*	0.11		0.03	0.03
*Lygosoma punctata*	0.26	0.22		0.04
*Asymblepharus himalayanus*	0.25	0.23	0.30	

Standard error estimate(s) are shown above the diagonal and were obtained by a bootstrap procedure (1000 replicates).

Presently, the lizard population is declining due to habitat fragmentation and habitat loss. Nowadays, natural habitats are regularly disappearing due to infrastructure development and human population growth. Remaining habitats are isolated from each other by urban development, roadways and agricultural fields.

Climate change, including higher temperatures, low soil moisture, longer dry seasons, and more variability in rainfall, can also affect the reptile population. Humans have altered natural disturbance, i.e. suppressing fire, controlling flood levels, and other means. These factors can be detrimental to amphibian and reptile populations. In many ecosystems, these disturbances help amphibians and reptiles to provide their needs of habitat and to maintain the various stages of vegetative succession (Amphibians and Reptiles 2007). If fire, flooding, or other natural disturbances are not present, then natural habitats become degraded and are less capable of supporting amphibian and reptile populations.

Introduction of invasive species is also a major factor for the declining population of reptiles. Native species have not evolved and survived with non-native species in their own area. For example, fire ants, introduced to the southern United States around 1918, prey on both the eggs and the young ones of reptiles. Fire ants are considered to be the primary cause of the extirpation of the Texas horned lizard from parts of its historic range. However, in India no such study, so far, has been done about the impact of non-native species on the distribution and survival of the herps.

## Conclusions

In India, Uttarakhand has the great biodiversity of flora and fauna. The current study only covers the small areas of Uttarakhand saurian diversity. During field survey, we collected the four skink species; after analysis and compression with publically available sequences, we concluded that the selected skink species 100% matched with respected species. Here, we trying to give the authentic genetic 12S rRNA reference database of selected skink species those highly useful for further study on phylogenetic and population genetic level. This study also gives the preliminary genetic authentication of four skink species in RTR, Uttarakhand (India).

It is necessary to establish and promote the use of such DNA profile database of mitochondrial DNA to other Uttarakhand population. This study also indicates that the exact population and species of skink are unidentified; therefore, we recommend the Uttarakhand skink population should be investigated further genetic study in combination with a good sampling strategy to investigate species biology (including patterns of genetic diversity, relatedness and population connectivity) and status for conservation program.

## References

[CIT0001] Aengals R, Kumar VMS, Palot MJ. 2011 Updated checklist of Indian reptiles. [accessed 2017 Jan 18]. http://zsi.gov.in/checklist/Checklist%20of%20 Indian%20Reptiles.pdf (online version).

[CIT0029] Amphibians and Reptiles. 2007. Natural Resources Conservation Service, Wildlife Habitat Council. Fish and Wildlife Habitat Management Leaflet. Number 35. [accessed 2017 Jul 25]. https://policy.nrcs.usda.gov/18528.wba

[CIT0003] Bahuguna A. 2010. Reptilia IT: Fauna of Uttarakhand, Vertebrates (Part-1) State Fauna Series 1.8, 445–503, ISBN 978-81-8171-249-3. Kolkata: Zoological Survey of India.

[CIT0002] Blyth E. 1854 Notices and descriptions of various reptiles, new or little-known. Part I. J Asiat Soc Bengal. 22:639–655.

[CIT0018] Checklist India Biodiversity Portal. 2015 List of reptiles encountered in and around Wildlife Institute of India, Dehradun Campus. [accessed 2017 Jan 12]. www.indiabiodiversity.org/checklist/show/361587

[CIT0019] Checklist India Biodiversity Portal. 2015 List of reptiles encountered in and around Forest Research Institute, Dehradun Campus. [accessed 2017 Jan 12]. www.indiabiodiversity.org/checklist/show/361617

[CIT0020] Checklist India Biodiversity Portal. 2015 List of reptiles encountered in and around Rajaji National Park, Dehradun Campus. [accessed 2017 Jan 12]. www.indiabiodiversity.org/checklist/show/49

[CIT0004] Chopra RN. 1995 Lizards (Reptilia: Sauria). Fauna of Rajaji National Park, Fauna of conservation Areas Calcutta, India: Zoological Survey of India; p. 87–90.

[CIT0005] Daniel JC. 2002 Indian Reptiles and Amphibians. p. 134–135.

[CIT0006] De Silva A, Vyas RV. 2010 *Eutropis carinata* The IUCN Red List of Threatened Species 2010. [accessed 2016 Dec 29]. 10.2305/IUCN.UK.2010-4.RLTS.T178621A7582836.en

[CIT0008] Dutta-Roy A, Singh M, Karanth KP. 2014 Phylogeny of the endemic skinks of the genus lygosoma (Squamata: Scincidae) suggests an *in situ* radiation. J Genet. 93:163–167. 24840833 10.1007/s12041-014-0321-z

[CIT0007] Dutta-Roy A, Singh M, Sirinivasulu C, Karanth KP. 2012 Phylogeny of the Asian Eutropis (Squamata: Scincidae) reveals an ‘into India’ endemic Indian radiation. Mol Phylogenet Evol. 63:817–824. 22406530 10.1016/j.ympev.2012.02.022

[CIT0009] Gmelin SG. 1799 Hist. Amphib. p. 197.

[CIT0010] Günther A. 1864 The reptiles of British India. London: Taylor & Francis.

[CIT0012] Hall TA. 1999 BioEdit: a user-friendly biological sequence alignment editor and analysis program for Windows 95/98/NT. Nucl Acids Symp Ser. 41:95–98.

[CIT0011] Hallowell E. 1857 Notice of some new and rare species of Scincidae in the collection of the Academy of Natural Sciences of Philadelphia. Trans Am Philos Soc (Philadelphia). 11:71–82.

[CIT0013] Hedges SB, Conn CE. 2012 A new skink fauna from Caribbean islands (Squamata, Mabuyidae, Mabuyinae). Zootaxa. 3288:1–244.

[CIT0014] Kimura M. 1980 A simple method for estimating evolutionary rates of base substitutions through comparative studies of nucleotide sequences. J Mol Evol. 16:111–120. 7463489 10.1007/BF01731581

[CIT0015] Kocher TD, Thomas WK, Meyer A, Edwards SV, Paabo S, Villablanca FX, Wilson AC. 1989 Dynamics of mitochondrial DNA evolution in animals: amplification and sequencing with conserved primers. Proc Natl Acad Sci USA. 86:6196–6200. 2762322 10.1073/pnas.86.16.6196 PMC297804

[CIT0016] Kumar VP, Kumar D, Goyal SP. 2014 Wildlife DNA forensic in curbing illegal wildlife trade: specie identification from seizures. Int J Forensic Sci Pathol. 2:38–42.

[CIT0017] Kumar S, Stecher G, Tamura K. 2016 MEGA7: Molecular Evolutionary Genetics Analysis version 7.0 for bigger datasets. Mol Biol Evol. 33:1870–1874. 27004904 10.1093/molbev/msw054 PMC8210823

[CIT0022] Mecke S, Doughty P, Donnellan SC. 2013 Redescription of *Eremiascincus fasciolatus* (Günther, 1867) (Reptilia: Squamata: Scincidae) with clarification of its synonyms and the description of a new species. Zootaxa. 3701:473–517. 26191600 10.11646/zootaxa.3701.5.1

[CIT0021] Murthy TSN. 2010 The reptile fauna of India. New Delhi: B.R. Publishing Corporation.

[CIT0023] Oppel M. 1811 Die Ordnungen, Familien und Gattungen der Reptilien, als Prodrom einer Naturgeschichte derselben [The orders, families and genera of the reptiles, as the prodrom of a natural history]. Munich: Joseph Lindauer.

[CIT0024] Rajpoot A, Kumar VP, Bahuguna A, Kumar D. 2016 Forensically informative nucleotide sequencing (FINS) for the first time authentication of Indian Varanus species: implication in wildlife forensics and conservation. Mitochondrial DNA Part A. 2017;27 DOI: 10.1080/24701394.2016.1202943 27838947

[CIT0026] Saitou N, Nei M. 1987 The neighbor-joining method: a new method for reconstructing phylogenetic trees. Mol Biol Evol. 4:406–425. 3447015 10.1093/oxfordjournals.molbev.a040454

[CIT0025] Schneider JG. 1801 Historiae Amphibiorum naturalis et literariae. Fasciculus secundus continens Crocodilos, Scincos, Chamaesauras, Boas. In: Schneider JG, editor. Pseudoboas, Elapes, Angues Amphisbaenas et Caecilias. Jena: Frommanni p. 1–290.

[CIT0028] Uetz P, Hošek J, Hallermann J. 1995–2013 The reptile database. [updated 2012 Dec 24; accessed 2013 Mar 25]. Electronic database accessible at http://www.reptiledatabase.org

[CIT0027] Venugopal PD. 2010 An updated and annotated list of Indian lizards (Reptilia: Souria) based on a review of distribution records and checklists of Indian reptiles. J Threat Taxa. 2:725–738.

